# Enhancing the Hydrolytic Activity of a Lipase towards Larger Triglycerides through Lid Domain Engineering

**DOI:** 10.3390/ijms241813768

**Published:** 2023-09-06

**Authors:** Laura Fernandez-Lopez, Sergi Roda, Ana Robles-Martín, Rubén Muñoz-Tafalla, David Almendral, Manuel Ferrer, Víctor Guallar

**Affiliations:** 1 Instituto de Catalisis y Petroleoquimica (ICP), CSIC, 28049 Madrid, Spain; l.fernandez.lopez@csic.es (L.F.-L.); d.almendral@csic.es (D.A.); 2 Department of Life Sciences, Barcelona Supercomputing Center (BSC), 08034 Barcelona, Spain; sergi.rodallordes@bsc.es (S.R.); ana.robles@bsc.es (A.R.-M.); ruben.munoz@bsc.es (R.M.-T.); 3 PhD Programme, Faculty of Pharmacy and Food Science, Universitat de Barcelona (UB), 08007 Barcelona, Spain; 4 Institution for Research and Advanced Studies (ICREA), 08010 Barcelona, Spain

**Keywords:** lipase, lid domain, protein engineering, rational design

## Abstract

Lipases have valuable potential for industrial use, particularly those mostly active against water-insoluble substrates, such as triglycerides composed of long-carbon chain fatty acids. However, in most cases, engineered variants often need to be constructed to achieve optimal performance for such substrates. Protein engineering techniques have been reported as strategies for improving lipase characteristics by introducing specific mutations in the cap domain of esterases or in the lid domain of lipases or through lid domain swapping. Here, we improved the lipase activity of a lipase (WP_075743487.1, or Lip_MRD_) retrieved from the Marine Metagenomics MarRef Database and assigned to the *Actinoalloteichus* genus. The improvement was achieved through site-directed mutagenesis and by substituting its lid domain (FRGTEITQIKDWLTDA) with that of *Rhizopus delemar* lipase (previously *R. oryzae*; UniProt accession number, I1BGQ3) (FRGTNSFRSAITDIVF). The results demonstrated that the redesigned mutants gain activity against bulkier triglycerides, such as glyceryl tridecanoate and tridodecanoate, olive oil, coconut oil, and palm oil. Residue W89 (Lip_MRD_ numbering) appears to be key to the increase in lipase activity, an increase that was also achieved with lid swapping. This study reinforces the importance of the lid domains and their amino acid compositions in determining the substrate specificity of lipases, but the generalization of the lid domain swapping between lipases or the introduction of specific mutations in the lid domain to improve lipase activity may require further investigation.

## 1. Introduction

Ester hydrolases (Enzyme Commission (EC) number 3.1.-.-) are enzymes responsible for the cleavage (under hydrolysis conditions) or formation (under synthetic conditions, e.g., transesterification) of ester bonds [1]. Specifically, those enzymes that carry out the hydrolysis of carboxylic esters into their respective acid and alcohol are known as carboxylic ester hydrolases (EC 3.1.1.1.-). This group is further divided into esterases (EC 3.1.1.1) and lipases (EC 3.1.1.3), which have been differentiated on the basis of their sequences and substrate specificity. Esterases hydrolyse water-soluble short-chain (<10–12 carbon atoms) acyl esters (e.g., *p*-nitrophenyl butyrate, *p*-NP butyrate) and are mostly inactive against water-insoluble long-chain (>12–18 carbon atoms) triacylglycerols (e.g., triolein), which, in turn, are specifically hydrolysed by lipases [2,3]. The difference between lipases and esterases has been a subject of continuing controversy, although most of the “true lipases” are proteins that carry out interfacial activation so that they attack insoluble drops of substrate [4].

Interfacial activation is the increase in enzyme activity when substrates change from being soluble in an aqueous medium to an aggregated state (such as an emulsion) due to insolubility [4,5]. From the discovery of this behaviour, the idea that there must be some structural explanation was postulated. In 1990, two lipases, the triacylglycerol lipase from the fungus *Rhizomucor miehei* [6] and the human pancreatic lipase [7], were crystallized and allowed the observation of a lid domain covering the active site. It was suggested that the lid plays a crucial role in interfacial activation, as its conformational change is needed to expose the active site to the entry of insoluble drops of substrates. This hypothesis was subsequently supported by the structural resolution of two lipase complexes, namely, that of *Rhizomucor miehei* lipase with *n*-hexylphosphonate ethyl ester [8] and that of the complex of human pancreatic lipase and procolipase with mixed micelles of phosphatidylcholine and bile salt [9]. This behaviour can be achieved due to the amphipathic nature of the lid domain, where the hydrophilic residues face the solvent and the hydrophobic residues face the catalytic site in the closed conformation. Once the enzyme opens the lid domain, the hydrophobic side helps in binding lipophilic substrates around the active site, and the lipase is the so-called active conformation [10,11]. However, it was later shown that not all lipases carried out this interfacial activation phenomenon. Examples are the cutinase from *Fusarium solani* [12], the pancreatic phospholipase from guinea pig [13], and the lipase from *Pseudomonas aeruginosa* [14], which lacked the lid. Additionally, the triacylglycerol lipase from *P. glumae* [15] and lipase B from *Candida antarctica* [16], both with a lid, do not show interfacial activation [17].

Recent studies have demonstrated that the presence of specific residues in the lid domain is closely correlated with the activity and specificity of lipases and the preference for substrates with a long carbon chain in the acyl region that are insoluble in water, such as triolein [18,19,20,21,22,23,24,25,26]. This has been widely proven by different studies on lid engineering that show how dramatically the activity and specificity of lipases can change [27,28,29]. Thus, the lid domain is an essential “hot spot” for tailoring lipases towards the user’s needs for many applications [30,31,32].

Here, we present the engineering of the lid domain of a lipase from the *Actinoalloteichus* genus (NCBI Accession Number: WP_075743487.1) to switch from the hydrolysis of small/medium-carbon chain triglycerides to large-carbon chain ones. This hydrolase was identified in the frame of sequence-based metagenomic bioprospecting for novel enzymes, particularly from marine environments, and it was selected as a target (i) because lipases from species of actinobacteria are known to be versatile and (ii) because of its good protein solubility upon expression in *Escherichia coli*. With the aim of increasing lipase activity, we used two alternative approaches. First, we computationally characterized the lid opening of the wild type, and by incorporating two mutations in the lid domain, activity against glyceryl tridecanoate (TriC_10:0_), as well as coconut, palm, and olive oil was achieved. Second, we used a lid swapping approach with which we also achieved a significant increase in the length of the triglyceride carbon chain being hydrolysed.

## 2. Results

### 2.1. Lipase Sequence-Based Metagenomic Bioprospecting

To discover novel lipases with activity towards large triglycerides, we screened the Marine Metagenomics MarRef Database [33] (https://mmp2.sfb.uit.no/, accessed on 3 August 2023); ca. 4.7 million protein coding sequences). The sequences were selected by querying the input sequences using DIAMOND BLASTP, using default parameters (percent identity >60%; alignment length > 70; e-value < 1 × 10^−5^) against the 392 amino acid lipase from *Rhizopus delemar* (UniProt accession number I1BGQ3; molecular mass, 42,138 Da; isoelectric point, 7.06), previously referred to as *R. oryzae* (acc. no. P61872). Although other lipases available in the databases could have been used as targets, we selected the one from *R. delemar* because (i) its catalytic centre is sheltered by an alpha-helix lid and shows significant activity towards large lipid substrates [34,35] (Table 1); (ii) it is a very versatile enzyme that has attracted the attention of several industrial enzyme producers due to its broad range of industrial applications for esterification, interesterification, and transesterification reactions; and, finally, (iii) it has a lid domain of similar size to that of the lipase identified and characterized in this study (16 amino acids). A total of 20 sequences were retrieved (e-values from 3.1 × 10^−14^ to 1 × 10^−5^ when compared to the lipase from *R. delemar*). One such sequence (GenBank accession number, WP_075743487), assigned to a bacterium of the genus *Actinoalloteichus*, was confirmed to encode a predicted full-length 271 amino acid-long hydrolase (e-value 2.53 × 10^−11^ and 33.6% similarity compared to lipase from *R. delemar*) with the needed catalytic residues and domains, and it was selected as a target for further investigation. Within the Marine Metagenomics MarRef Database, the sequence WP_075743487 originated from a microbiome isolated from marine sponges in a sea coast area (Norway: Trondheim fjord; ENA BioSample accession SAMN03339750; ENA BioProject accession PRJNA275157) [36].

### 2.2. Synthesis, Expression, Purification and Characterization of Lip_MRD_

Once identified, the 271 amino acid sequence encoding the wild-type enzyme (GenBank acc. Nr. WP_075743487; molecular mass, 30,173 Da; isoelectric point, 5.31) was used as a template for gene synthesis, which was performed as detailed in the Materials and Methods. After synthesis, a 294 amino acid sequence was obtained encoding an enzyme with a molecular mass of 32,698 Da and an isoelectric point of 5.40. The soluble *N*-terminal hexahistidine (His_6_)-tagged protein was produced and purified (>98% using SDS–PAGE analysis; Figure S1) after binding to a Ni-NTA His-Bind resin. From now on, this enzyme is referred to as Lip_MRD_ (Lip refers to lipase; MRD refers to the MarRef Database).

The hydrolytic activity of purified protein (Lip_MRD_) was initially evaluated against a series of triglycerides with different carbon chain lengths, namely, glyceryl tripropionate (TriC_3:0_), tributyrate (TriC_4:0_), trioctanoate (TriC_8:0_), and tridecanoate (TriC_10:0_), as well as coconut oil, which is typically dominated by medium-carbon chain triglycerides of lauric acid (TriC_12:0_), myristic acid (TriC_14:0_), palmitic acid (TriC_16:0_), olive oil (or triolein), which is mostly composed of long-carbon chain triglycerides of oleic acid (TriC_18:1_), and palm oil, which is dominated by long-carbon chain triglycerides of palmitic acid (TriC_16:0_) and stearic acid (TriC_18:0_). The protein was found to be active against short-carbon chain (TriC_3:0_ and TriC_4:0_) to medium-carbon chain (TriC_10:0_ and coconut oil) triglycerides, with specific activities ranging from 50 to 3230 units/g protein, measured at pH 8.0 and 30 °C (Table 1).

**Figure 1:**
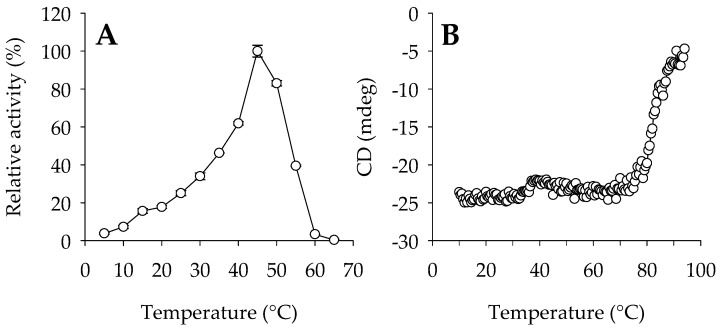
Thermal characteristics of Lip_MRD_. (**A**) Temperature profile. The effect of temperature was determined following the hydrolysis of the model ester *p*-NP butyrate at 348 nm in 40 mM 4-(2-hydroxyethyl)-1-piperazineethanesulfonic acid (HEPES) buffer at pH 7.0. Values are plotted as the mean of triplicate results (n = 3) with the reported standard deviation (SD) calculated using the STDEV.S function in Excel 2019, with control reactions (no enzyme added) considered. (**B**) The thermal denaturation curve at pH 7.0, as determined by CD spectroscopy measuring the ellipticity changes at 220 nm obtained at different temperatures at a rate of 0.5 °C per min. In A, the maximal activity was defined as 100% (597.4 ± 1.8 units/mg), and relative activity is shown as the percentage of maximal activity (mean ± SD of triplicates) determined under the conditions described in the Section 4. The figure was created using SigmaPlot 14.5. The raw data can be found in the Supplementary Material (Raw Dataset).

**Figure 2:**
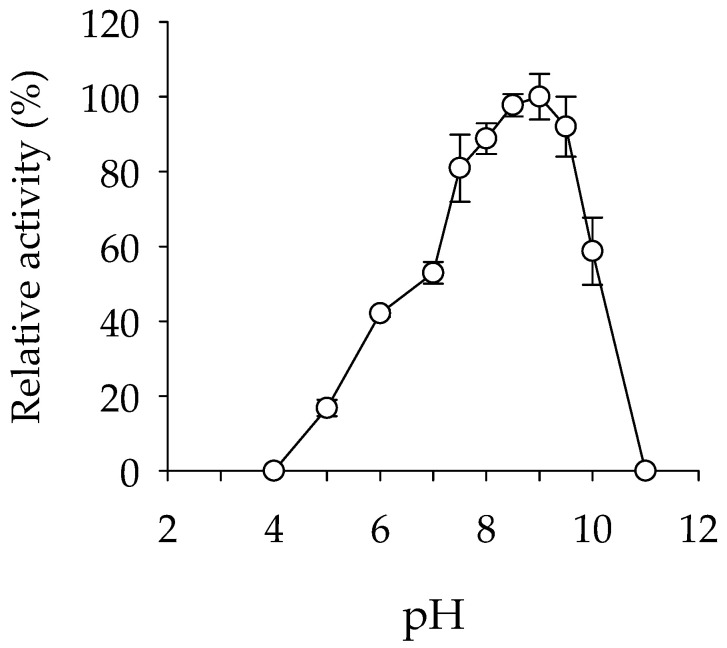
Optimal pH for Lip_MRD_ lipase activity. The effect of pH was determined at 30 °C following the hydrolysis of the model ester *p*-NP butyrate at 348 nm in 50 mM Britton-Robinson (BR) buffer at pH 4.0–11. Values are plotted as the mean of triplicate results (n = 3) with the reported SD calculated using the STDEV.S function in Excel 2019, with control reactions (no enzyme added) considered. The maximal activity was defined as 100% (384.0 ± 23.4 units/mg), and relative activity is shown as the percentage of maximal activity (mean ± SD of triplicates) determined as described in the Materials and Methods. The figure was created using SigmaPlot 14.5. The raw data can be found in the Supplementary Material (Raw Dataset).

The enzyme showed maximal activity at 45 °C, retaining more than 35% of the maximum activity at 30–55 °C (Figure 1A). Analysis by circular dichroism (CD) spectroscopy revealed that the enzyme showed a sigmoidal curve with two transitions, one with a denaturation temperature (*T*_d_) of 46.3 ± 1.8 °C and a second at 82.4 ± 0.2 °C (Figure 1B). We checked whether Lip_MRD_ could have a multimeric structure with GalaxyHomomer [40], a protein homo-oligomer structure prediction method, and a plausible dimeric structure was obtained using the sequence-based method (Figure S2). Thus, the presence of these two phases might be due to a multimeric enzyme structure that is disturbed by the thermal conditions. Thermal denaturation of the protein is a condition in which the unique 3D structure of a protein is disturbed, and it is possible that due to changes in temperature, pH, or other chemical conditions, the hydrogen bonds present in the proteins may also be disturbed. Therefore, we cannot rule out that the two phases could also exist under our assay conditions (no salt added and pH 7.0) due to a rapid change in protein conformation, yet to be determined, that impairs but does not inactivate the enzyme, followed by a slow change in protein multimeric structure that results in complete inactivation. Finally, its optimal pH for activity is 9.0, and it retains more than 50% of the maximum activity at pH values from 7.0 to 10.0 (Figure 2).

The hydrolase did show maximal activity at a pH of 8.5 and a temperature of 30 °C, values similar to those of the lipase from *R. delemar* used as a target for bioprospecting [34,35,41]. The substrate specificity of the lipase from *R. delemar* was further evaluated under the same assay conditions used to test Lip_MRD_. For that, we used the commercial preparation Addzyme RD (Evoxx Technologies GmBH). As shown in Table 1, under our assay conditions, this preparation was most effective for hydrolysing TriC_8:0_ but also converted larger triglycerides such as triolein (specific activities ranging from 590 to 13,440 units/g protein); this is in agreement with the results of previous studies in which this enzyme was tested with triglycerides from tributyrin to triolein [34,35,41]. Note that the hydrolase Lip_MRD_ and the lipase Addzyme RD have entirely different specificities with regard to their preference for shorter or larger triglycerides, respectively. This may be due to the low similarity between their sequences (approximately 33%) and the differences in the architecture of their active sites and the structure of their lid domains; the latter point will be discussed below.

### 2.3. Molecular Simulations for Improving Lipase Activity

**Figure 3:**
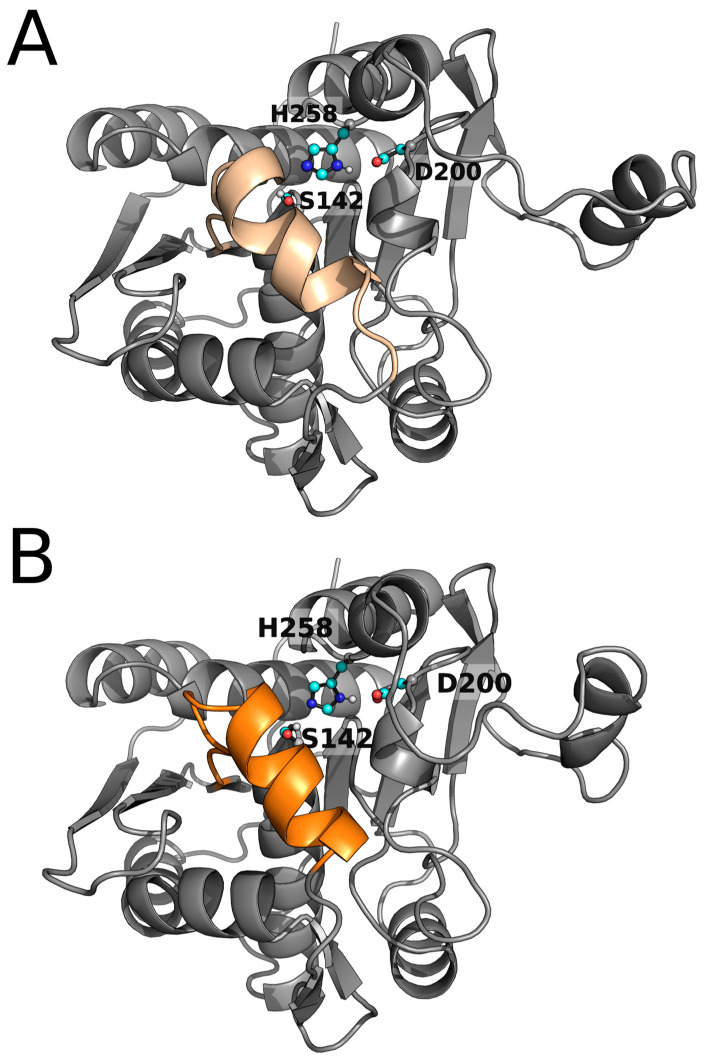
AlphaFold 3D models of Lip_MRD_ (**A**) and Lip_MRDlid_ (**B**) highlighting the catalytic triad (with C atoms coloured in deep-blue and red) and the lid domain (coloured in wheat and orange).

As shown in Table 1, the enzyme Lip_MRD_ was active towards small-carbon chain triglycerides (glyceryl tributyrate) but had no activity against bulkier triglycerides under our assay conditions. Since engineering of the lid domain can lead to drastic changes in the activity and specificity of lipases [19], we visually inspected this structural motif in Lip_MRD_ (Figure 3) to introduce mutations that may allow the hydrolysis of bulkier triglycerides. For that purpose, we first computationally studied the lid opening of the wild-type enzyme and that of the *R. delemar* lipase as a control, and we then tried to design a mutant based on that analysis.

In brief, as detailed in the Materials and Methods, Lip_MRD_ was computationally studied by first obtaining its AlphaFold model and then by preparing and protonating it by Protein Preparation Wizard under conditions similar to those used in our experimental setup and by performing Monte Carlo simulations using Protein Energy Landscape Exploration (PELE).

**Figure 4:**
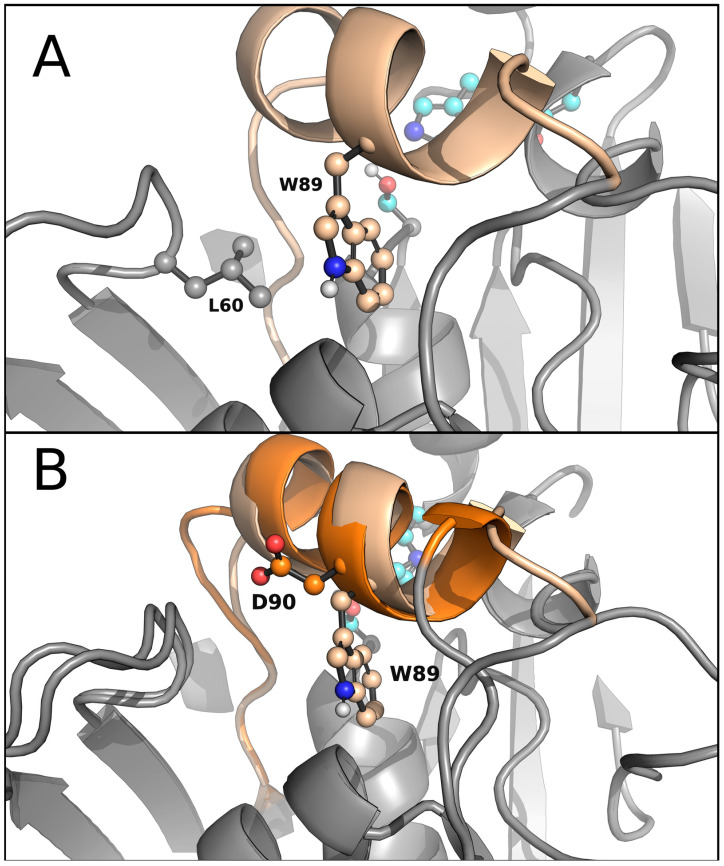
AlphaFold 3D models of Lip_MRD_ and Lip_MRDlid_. (**A**) AlphaFold 3D model of Lip_MRD_ highlighting the positions of W89 and L60 in the lid domain (coloured in wheat). (**B**) AlphaFold 3D models of Lip_MRD_ and Lip_MRDlid_ depicting the absence of the bulky W89 residue in the lid domain of the mutant (coloured in wheat and orange).

**Figure 5:**
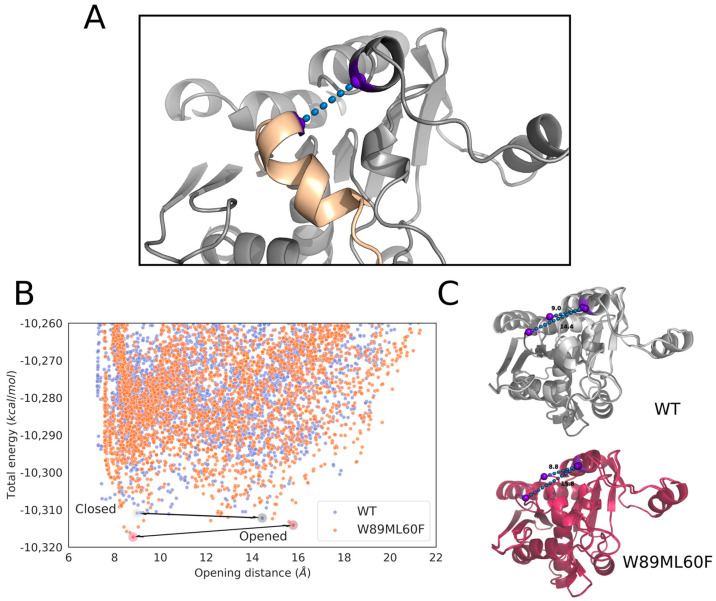
Opening of the lid domain of Lip_MRD_ through Monte Carlo simulations. (**A**) 3D representation of the opening distance in the Lip_MRD_ system. (**B**) Scatter plots of the opening distance against the total energy of the system. The energy profiles were created with the Matplotlib library [46]. (**C**) 3D displays of the opened and closed metastable states of both the wild-type enzyme and the double mutant.

The original lid domain (FRGTEITQIKDWLTDA) of Lip_MRD_ seemed to have a tryptophan residue at the inner side, hindering it from fully opening to bind medium- and long-carbon chain triglycerides (Figure 4 and Figure S3), in agreement with experimental data (Table 1). This amino acid is absent in the lid domain (FRGTNSFRSAITDIVF) of *R. delemar* lipase (Figure 4). We performed a Monte Carlo simulation with PELE software (version rev12360) [42,43] to test this hypothesis. The simulation consisted of the lipase being solely perturbed by a vector between the ɑ carbons of a residue in the lid domain (I83 for the Lip_MRD_ enzyme and F209 for the *R. delemar* lipase) and a residue in the loop in front of it (L255 for the Lip_MRD_ enzyme and I377 for the *R. delemar* lipase), forcing the opening of the lid domain. After this directed perturbation of the lid domain, the system was minimized, and the step was accepted or rejected based on the Metropolis criterion. Moreover, the sense of the mentioned vector was changed every 100 steps to study the motion related to the opening and closure of the lid domain. This back-and-forth motion allowed us to find the metastable opened and closed states during the calculation. Measuring the difference in a distance that can represent the opening and closure of the lid domain of the opened and closed states between systems can tell us the size of the substrates that each enzyme could bind and hydrolyse. In this study, the distance used was the described vector to move the lid domain (Figure 5).

The results of the simulations showed that the wild-type enzyme had a closed metastable state at 8.99 Å and an open metastable state at 14.43 Å, meaning that the lid domain opens up to 5.44 Å (Figure 5). On the other hand, the *R. delemar* lipase had a closed metastable state at 7.71 Å and an opened state at 15.30 Å, showing a difference in the opening distance of 2.14 Å (Figure S4). Thus, we created a variant aimed at further opening the lid domain of the lipase. The designed mutant replaced W89 with a less bulky residue, methionine, but this was still a reasonable change according to the BLOSUM62 matrix. To compensate for the increase in volume in the closed conformation of the enzyme due to the mentioned mutation and to prevent the access of water molecules to the active site, L60, a residue not placed in the lid domain (Figure 4) was mutated to phenylalanine. Then, the same type of simulation was performed on the double mutant (W89M/L60F). The results gave a closed metastable conformation (at 8.81 Å) similar to that of the wild-type enzyme but a more open conformation (at 15.78 Å) (Figure 5). Thus, the difference in the opening distance between the double mutant and the wild-type was approximately 1.5 Å, meaning that the variant appeared to be opening more, potentially allowing it to fit bulkier triglycerides in the active site.

**Figure 6:**
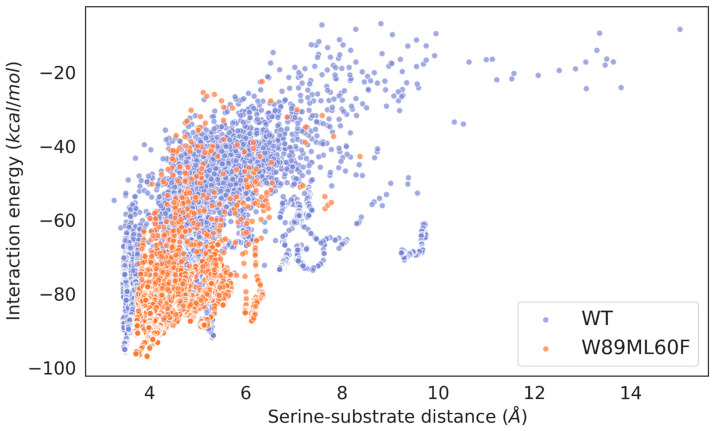
Scatter plots of the serine-substrate (nucleophilic O of the catalytic Ser residue and ester Cs of the substrate) distance against the interaction energy of the wild-type enzyme and the double mutant. The energy profiles were created with the Matplotlib library [46].

To confirm this hypothesis and evaluate to what extent the mutations and the different lids affect the catalytic binding of bulkier triglycerides, local explorations of the binding of triolein (a long-carbon chain triglyceride) in the active state of the wild-type enzyme (Lip_MRD_) and its double mutant (W89M/L60F) as well as the *R. delemar* lipase were performed. The ligand was undockable in the open conformation of the wild-type enzyme (in agreement with the difficulties of the wild-type enzyme in hydrolysing long-carbon chain triglycerides), so we had to migrate a triolein molecule from the solvent to the active site with AdaptivePELE [42,43,44] with a bias that minimizes the distance between the substrate and the catalytic serine residue. The simulation successfully gave catalytic binding positions of the substrate around the active site (Figure S5). On the other hand, the ligand was easily docked with Glide software (version 95128) [45] on the open conformations of the double mutant and the *R. delemar* lipase (Figure S6). The induced-fit simulations showed that the substrate spent more time bound in a catalytic conformation in the double mutant and the *R. delemar* lipase compared to the wild-type enzyme (Figure 6 and Figure S7). The wild-type enzyme only had ~56% of PELE poses inside the active site (with the serine-substrate distance equal to or lower than 5 Å), while the double mutant and the *R. delemar* lipase had up to ~82% and 99% of poses within the active site, respectively. The number of catalytic events, poses where one of the ester C atoms from the substrate molecule is 4 Å from the nucleophilic O of the catalytic serine residue and the H-bonds of the catalytic triad have appropriate distances, is similar in the double mutant, with 1696 (and 10.412% of all accepted PELE steps), and the wild-type enzyme, with 2023 (and 12.687% of all accepted PELE steps) such events. The *R. delemar* lipase showed more predicted catalytic events than the Lip_MRD_ wild-type enzyme with 33,604 (and 91.586% of all accepted PELE steps). The higher number of such catalytic events agrees with the higher catalytic activity of *R. delemar* lipase compared with Lip_MRD_, as determined experimentally (see Table 1).

The sequence encoding Lip_MRD_ with the two mutations, W89M and L60F (Lip_MRDW89M/L60F_), was synthesized as for the wild-type. After synthesis, a 294 amino acid-long sequence was obtained encoding an enzyme with a molecular mass of 32,677 Da and an isoelectric point of 5.40. The mutant was expressed, purified, and characterized as the wild-type protein. The protein was found to be active against small- to large-length triglycerides, including trioctanoate (TriC_8:0_), tridecanoate (TriC_10:0_), coconut oil, palm oil, and olive oil (Table 1); note that the last two substrates were not hydrolysed by the wild-type protein. Specific activities ranged from 550 (for TriC_3:0_) to 13,800 (for TriC_8:0_) units/g protein, showing the ability to hydrolyse triglycerides as large as olive oil (1760 units/g) and palm oil (1050 units/g). These results agree with the computational analysis and the role of residue W89, located in the original lid domain, in the substrate specificity and access of bulkier triglycerides to the active site.

### 2.4. Molecular Simulations: Lid Swapping

As a complementary strategy to further improve the capacity of the lipase to hydrolyse bulkier triglycerides, and based on the results found by mutating a lid residue, we performed lid swapping. The lid domain (FRGTEITQIKDWLTDA) of Lip_MRD_ was replaced by that (FRGTNSFRSAITDIVF) of *R. oryzae* lipase, which was used as a template for screening Lip_MRD_ in the Marine Metagenomics MarRef Database and shares 33.2% sequence identity in a local sequence alignment and an RMSD of ~3 Å with its crystal structure [47] (PDB code: 1TIC, against Lip_MRD_’s AlphaFold model). This enzyme shows an optimum pH of 8.5 and an optimum temperature of 30 °C and can accept short (TriC_4:0_), medium (TriC_8:0_), and large (triolein) triglycerides [34,35,41] (see Table 1). Such substrate specificity of lipases has been commonly associated with the presence of a lid domain and the residues (e.g., hydrophobic amino acids) that conform to it [47,48]. The differences in the hydrophobic characteristics of the lid domains of the two enzymes raise the question of whether lid swapping may improve the lipase activity of Lip_MRD_.

First, we performed the same computational analysis for the new lid-swapped mutant, named Lip_MRDlid_ (Figure 3 and Figure 4). The lid opening type of simulation for the Lip_MRDlid_ variant showed a closed metastable conformation at 9.40 Å and a more opened conformation at 19.61 Å, meaning that this variant had the highest opening distance of all interrogated species (Figure S8). The induced-fit simulation of the lid-swapped mutant showed that the substrate stayed bound in a catalytic conformation ~99% of the time (Figure S9). Moreover, the number of catalytic events was 30,392 (and 91.295% of all accepted PELE steps), the highest compared to the wild-type enzyme and the double mutant, and with similar values to the *R. delemar* lipase.

The engineered Lip_MRDlid_ variant was then synthesized as for the wild-type. After synthesis, a 294 amino acid sequence was obtained, encoding an enzyme with a molecular mass of 32,635 Da and an isoelectric point of 5.56. The mutant (N-terminal His_6_-tagged) was expressed, purified, and characterized as for the wild-type protein. The protein was found to be active against all triglycerides tested, with specific activities ranging from 630 (for TriC_3:0_) to 2330 (for TriC_8:0_) units/g protein, being able to hydrolyse triglycerides as large as olive oil (1210 units/g) and palm oil (1290 units/g) to an extent similar to that of the Lip_MRDW89M/L60F_ mutant (Table 1).

It should be noted that the two mutants designed in this study showed a preference for long triglycerides similar to that of the model *R. delemar* lipase (Table 1), although the specific activities are not comparable to the latter values derived from a nonpure commercial sample (Addzyme RD).

## 3. Discussion

Understanding the mechanisms that modulate the substrate specificity of ester hydrolases, both esterases and lipases, and, in particular, the increase in lipase activity in this type of enzyme, has been the subject of analysis both in native enzymes and in mutants designed by protein engineering techniques [19,20,21,22,23,24,25,26]. In this context, many ester hydrolases present a lid domain (in the case of lipases) [10,11,27,28,29] or a cap domain (in the case of some esterases) [48] whose function is to allow the entry of substrates to the active site. This is known as interfacial activation in the case of lipases, with open and closed forms depending on the displacement of this lid [10,11,27,28,29]. It has been observed that the lid described in lipases and the cap domain of esterases have very similar topology, although in the latter, no biological functionality has been observed. Nevertheless, high flexibility of the *N*-terminal end has been observed [49]. Although it is not possible to strictly speak of open and closed forms in esterases, in the specific case of some members of the family IV esterases, the opening of the cap domain seems to be a prerequisite for the entry of substrates into the active centre, something that is reminiscent of the role of the lid domain in lipases. This occurs, for example, because of the presence in the cap domain of residues that could act as hinges in the opening of the *N*-terminal part of the cap to facilitate the entry of bulky substrates into the active centre [50,51,52,53]. Because lipases and esterases come from a common ancestor, the high flexibility of the cap domain observed in the latter could be reminiscent of the interfacial activation of lipases.

Using protein engineering to alter the amino acids of the cap domain in a single esterase and thus modulate the mobility of the cap domain has allowed us to shape the entry of bulkier substrates into the active centre and therefore alter the substrate specificity of the esterase [48]. These advances have not occurred at the same level in lipases [27,29]. Indeed, lipases with different residues and characteristics (e.g., hydrophobicity) or the same lipase with specific changes introduced in the lid can show very different activity profiles and specificity. In this study, we have gone further and approached improving the lipase activity of a lipase, retrieved by metagenomics bioprospecting, through computationally predicted mutagenesis and lid swapping. Specifically, by introducing a double mutant, we significantly increased the size of accepted substrates as well as the activity on medium-carbon chain substrates. Moreover, we changed the lid of a lipase with little lipase activity to the lid of another enzyme with reported lipase activity. The results provided in this study demonstrate unequivocally that this method produces a lipase with better characteristics in terms of broadening the preference or ability to hydrolyse longer and insoluble substrates. Validation of computation predictions reinforces the idea that the differences in the presence of key residues between the original and the swapped lid domains play a major role in improving lipase activity. It cannot be ruled out that other factors derived from the incorporation of the new lid domain may contribute to the increase in the lipase activity of the originating enzyme, as shown by the improved lipase activity of the Lip_MRD*lid*_ mutant compared to the Lip_MRDW89M/L60F_ mutant.

## 4. Materials and Methods

### 4.1. Source and Production of Lip_MRD_, Lip_MRDlid_ and Lip_MRDW89M/L60F_

The sequences of Lip_MRD,_ Lip_MRDlid_*,* and Lip_MRDW89M/L60F_ were synthesized by GenScript Biotech (GenScript Biotech, EG Rijswijk, The Netherlands) and codon-optimized to maximize the expression in *E. coli*. The genes were flanked by BamHI and HindIII (stop codon) restriction sites and inserted into a pET-45b(+) expression vector with an ampicillin selection marker (GenScript Biotech, Rijswijk, The Netherlands), which was further introduced into *E. coli* BL21(DE3). This plasmid, which was introduced into *E. coli* BL21(DE3), supports the expression of *N*-terminal His_6_-fusion proteins, with the final amino acid sequence of the synthetic protein being MAHHHHHHVGTGSNDDDDKSPDP-X (where X corresponds to the original sequence of the target enzyme). The soluble *N*-terminal His_6_-tagged proteins were produced and purified (>98% purity, as determined by SDS–PAGE analysis using a Mini PROTEAN electrophoresis system, Bio-Rad, Madrid, Spain) at 4 °C after binding to a Ni-NTA His-Bind resin (Merck Life Science S.L.U., Madrid, Spain), as previously described [39], and stored at −20 °C until use at a concentration of 1.5 mg/mL in 40 mM HEPES buffer (pH 7.0). Approximately 10–14 mg of purified proteins were obtained on average from a 1-L culture.

This work reinforces that the presence and movement of the lid domain are key for lipase activity. Having the His6-tag on the protein may influence lid movement and therefore activity. Although we did not produce a synthetic variant of the protein without a His6-tag to prevent any possible effect due to the tag, the distance from the *N*-terminus to both the lid domain and the catalytic triad in the AlphaFold model suggests no influence (Figure S10).

### 4.2. Source of R. delemar Lipase

Lipase from *R. delemar* (Addzyme RD) was kindly provided by Evoxx Technologies GmBH (Monheim am Rhein, Germany). Prior to use, a stock solution of 1 mg/mL in 40 mM HEPES buffer (pH 7.0) was prepared and used for activity tests.

### 4.3. Substrate Specificity

The enzymes (20 µg/mL) were incubated with a stock solution of each of the target esters, TriC_8:0_ (ref. T9126), TriC_10:0_ (ref. CRM44897), coconut oil (re. C1758), palm oil (ref. 70905), and olive oil (ref. O1514) (all provided by Merck Life Science S.L.U., Madrid, Spain), in 100 µL of (4-(2-hydroxyethyl)-1-piperazinepropanesulfonic acid (EPPS) buffer, 5 mM, pH, 8.0; T, 30 °C. The reactions were allowed to proceed in 2-mL safe-lock Eppendorf^®^ polypropylene tubes (ref. 0030 120.094, Greiner Bio-One GmbH, Kremsmünster, Austria) in a thermoshaker (model Thermomixer comfort, Eppendorf AG, Hamburg, Germany) at 950 rpm. After 30 min of reaction, hydrolysis was measured by using the NEFA Kit (FUJIFILM Wako Chemicals Europe GmbH, Neuss, Germany) following the manufacturer’s instructions. Briefly, 10 µL of the reaction solution was mixed with 100 µL of NEFA solution 1 in a 96-well plate (ref. 655801, Greiner Bio-One GmbH, Kremsmünster, Austria). Following 6 min of incubation at 37 °C, 50 µL of NEFA solution 2 was added, and after 6 min of incubation at 30 °C, the samples’ absorbance was measured at 550 nm using a Synergy HT Multi Mode Microplate Reader (BioTek Instruments, Winooski, VT, USA) with Gen5 2.00 software. Stock solutions were prepared at concentrations of 282.41 mg/mL for TriC_8:0_, 332.92 mg/mL for TriC_10:0_, 460 mg/mL for coconut oil, 431 mg/mL for palm oil and 431 mg/mL for olive oil in dimethyl sulfoxide (Merck Life Science S.L.U., Madrid, Spain); this corresponds to 0.6 M of all esters. The final concentrations in the reaction assays were 11.29 mg/mL for TriC_8:0_, 13.32 mg/mL for TriC_10:0_, 18.4 mg/mL for coconut oil, and 17.24 mg/mL for palm oil and olive oil. The activity was calculated by determining the absorbance per minute and by using a NEFA standard (oleic acid, ref. 29124-2, Merck Life Science S.L.U., Madrid, Spain) for calibration. One unit of enzyme activity was defined as 1 µmol of acid produced per minute under the assay conditions.

The activity towards TriC_3:0_ (ref. W328618) and TriC_4:0_ (ref. W222305), whose hydrolysis cannot be followed by the NEFA-Kit, was determined using a pH indicator (Phenol Red^®^) assay [37,38,39]. In brief, reactions were performed as follows: [enzyme], 2.8–45.5 µg/mL (depending on the enzyme); [TriC_3:0_ or TriC_4:0_], 4.5 mg/mL; reaction volume, 40 µL (4-(2-hydroxyethyl)-1-piperazinepropanesulfonic acid, EPPS buffer, 5 mM, phenol red (extinction coefficient of phenol red, 8450 M^−1^ cm^−1^), 0.45 mM, pH 8.0; T, 30 °C; assay format, 384-well plates (ref. 781162, Greiner Bio-One GmbH, Kremsmünster, Austria); and assay wavelength, 550 nm. Datasets were collected with a Synergy HT Multi-Mode Microplate reader (with Gen5 2.00 software Biotek Instruments, Winooski, VT, USA), with values obtained from the best linear fit using Excel 2019. In all cases, the activity was calculated by determining the absorbance per minute from the generated slopes [39]. One unit (U) of enzyme activity was defined as the amount of enzyme required to transform 1 µmol of substrate in 1 min under the assay conditions.

All reactions were performed in triplicate (n = 3) with control reactions (no enzyme added) and background signals considered, and the activity was calculated by determining the absorbance per minute from the generated slopes, as previously reported [39]. The threshold for activity was defined as at least twofold the background signal [54].

### 4.4. pH and Thermal Profiles

The hydrolysis of the model ester *p*-NP butyrate (ref. N-9876; Merck Life Science S.L.U., Madrid, Spain) was assessed by monitoring the continuous production of 4-nitrophenol at 348 nm (pH-independent isosbestic point, ε = 4147 M^−1^ cm^−1^) using 0.2 µg of total protein, as previously reported [39]. In all cases, a Synergy HT Multi-Mode Microplate Reader with Gen5 2.00 software (Biotek Instruments, Winooski, VT, USA) was used. The effect of the pH on the activity was evaluated in 50 mM BR buffer at pH 4.0–10.0. Note that the BR buffer consists of a mixture of 0.04 M H_3_BO_3_, 0.04 M H_3_PO_4_, and 0.04 M CH_3_COOH that was titrated to the desired pH with 0.2 M NaOH. Similar assay conditions were used to assay the effects of temperature on *p*-NP butyrate hydrolysis, but in this case, the reactions were performed at 40 mM HEPES buffer pH 7.0. The activity was calculated by determining the absorbance per minute from the generated slopes, as previously reported [39], with all reactions performed in triplicate (n = 3) with control reactions and background signals considered, as detailed above.

CD spectroscopy was used to determine the thermal denaturation profile. CD spectra were acquired between 190 and 270 nm with a Jasco J-720 spectropolarimeter (Jasco Inc., Tokyo, Japan) equipped with a Peltier temperature controller employing a 0.1-mm cell at 25 °C. Spectra were analysed and processed with a Spectra Manager software (Jasco Inc., Tokyo, Japan), and the denaturation temperature (*T*_d_) values were determined at 220 nm between 10 and 85 °C at a rate of 30 °C per hour in HEPES buffer 40 mM, pH 7.0. A protein concentration of 1.0 mg ml^−1^ was used. The *T*_d_ (and standard deviation of the linear fit) was calculated by fitting the ellipticity (mdeg) at 220 nm at each of the different temperatures using a 5-parameter sigmoid fit with SigmaPlot 14.0 software.

In this work, HEPES and BR buffers were selected for the activity assay. Although we did not anticipate any effect of the buffers in the results, since a prevalent feature of hydrolases is the possibility to show acyltransferase activity with alcohols and amines as acceptors, care must be taken in buffer selection. Phosphate buffer is generally preferable because it cannot act as an acyl acceptor.

### 4.5. Protein and Chemical Preparation for In Silico Analysis

The lipase from the *Actinoalloteichus* genus model was obtained using AlphaFold [55]. Then, the obtained AlphaFold model was prepared and protonated at pH 8.0, the pH at which the experimental assays were performed, using the Protein Preparation Wizard. The ester compound used was triolein. All substrates were modelled using the OPLS2005 force field [56]. The atomic charges of triolein were calculated with Jaguar [57] using density functional theory with a B3LYP-D3 exchange-correlation functional and the polarized double-zeta (pVDZ) basis set.

### 4.6. PELE Simulations

PELE (version rev12360) was used to model the opening of the lid domain in the studied lipase, as well as the binding of triolein to the lipase catalytic site. PELE is a Monte Carlo (MC)-based algorithm coupled with protein structure prediction methods [42,43]. This MC method starts with the sampling of different microstates by applying small perturbations (translations and rotations) on the ligand. Then, the flexibility of the protein is considered by applying normal modes through the anisotropic network model (ANM) approach [58]. Once the system has been perturbed, side chains of the residues near the ligand are sampled with a library of rotamers to avoid steric clashes. Finally, a truncated Newton minimization with the OPLS2005 force field [56] is performed, and the new microstate is accepted or rejected according to the Metropolis criterion. The Variable Dielectric Generalized Born Non-Polar (VDGBNP) implicit solvent [59] was applied to mimic the influence of water around the protein.

## 5. Conclusions

This work provides a rational-based protein engineering approach to improve the capacity of lipases to hydrolyse large water-insoluble triglycerides. This was achieved by investigating a lipase isolated from the Marine Metagenomics MarRef Database, which contains a lid domain but was only capable of hydrolysing triglycerides up to tridecanoate (TriC_10:0_) and showed slight activity towards coconut oil. There are examples of lipase engineering by lid swapping, which results in altered substrate specificity [27,28,29], and we therefore engineered a mutant of this hydrolase in which its lid domain was replaced by that of another lipase capable of degrading large-carbon chain triglycerides. The resulting lid-swapped construct could increase the range of triglycerides hydrolysed up to palm oil. Thus, lid swapping can help to tune the substrate profiles of lipases towards large-chain fatty esters beyond the tunnel and active site engineering [48] and specific mutations at the lid domain [23]. The results of the present study are complementary to those of a study that has been conducted to convert a lipase into an esterase capable of better hydrolysing water-soluble substrates by modifying the lid region [28] or to improve the lipase activity of lipases [27,28,29]. We will continue to explore the biocatalytic potential of lipases other than those investigated herein by using this approach. That said, whether the strategy provided here is transferable to other lipases is something to be investigated in the future; the approach may not be generalizable. Engineering the same lipase with different lid domains or incorporating the same lid domain into different lipase scaffolds may help to explore the versatility and potential of these types of lipase designs. This work reinforces the importance of the lid domain and its amino acid composition in determining and promoting lipase activity. The possibility of shortening, lengthening, or removing the lid domain may also emphasize the necessity of the lid domain in the enzyme investigated herein.

## Figures and Tables

**Table 1:** Substrate specificity of enzyme variants investigated in this study.

Triglyceride	Spec. Act. (Units/g) ^1,2^ Lip_MRD_	Spec. Act. (Units/g) ^1^ Addzyme RD	Spec. Act. (Units/g) ^1,2^ Lip_MRDlid_	Spec. Act. (Units/g) ^1,2^ Lip_MRDW89M/L60F_
TriC_3:0_	960 ± 20	590 ± 80	630 ± 20	550 ± 70
TriC_4:0_	1080 ± 10	1080 ± 90	780 ± 70	640 ± 10
TriC_8:0_	3230 ± 10	13,440 ± 60	2330 ± 90	13,800 ± 290
TriC_10:0_	580 ± 10	9850 ± 350	2220 ± 60	9220 ± 320
Coconut oil	50 ± 10	6630 ± 180	1460 ± 170	1860 ± 370
Palm oil	n.d. ^3^	1300 ± 250	1290 ± 210	1050 ± 20
Olive oil	n.d. ^3^	3680 ± 270	1210 ± 20	1760 ± 210

^1^ Specific activity (unit/g; mean ± SD of triplicates calculated using Excel 2019 STDEV.S function) for 7 model substrates. Activity was determined at 30 °C and pH 8.0. In brief, the activity towards TriC_3:0_ and TriC_4:0_ was determined using a pH indicator (Phenol Red^®^) assay [37,38,39]; for the other substrates, activity was evaluated by the NEFA-Kit. For details, see the Materials and Methods section. The raw data can be found in the Supplementary Material (Raw Dataset). ^2^ The fact that Lip_MRD_ has an initial *T*_d_ of 46.3 ± 1.8 °C suggests that the enzyme does not denature at the optimal temperature (45 °C) under our assay conditions (using *p*-NP butyrate and 1–7 min reaction time), although under other assay conditions (e.g., assay time) where stability may play a role, the optimal temperature plot may differ. This is why, for the determination of specific activity towards triglycerides, where a 30 min reaction assay was used, a temperature of 30 °C was set up to ensure protein stability during the assay. ^3^ No activity was detected (n.d.) under our assay conditions (30 min reaction time, 30 °C and pH 8.0) or after 24 h of incubation. It is possible that under other reaction conditions, including higher temperature and incubation times, some conversion may be observed.

## Data Availability

The raw data supporting the reported validation results can be found in the Supplementary Material (Raw Dataset).

## Supplementary Materials

The following supporting information can be downloaded at https://www.mdpi.com/article/10.3390/ijms241813768/s1.
