# Supplementary material for: Enhancing the Hydrolytic Activity of a Lipase towards Larger Triglycerides through Lid Domain Engineering
Source: Int J Mol Sci. 2023 Sep 6;24(18):13768. doi: 10.3390/ijms241813768 (PMC10530837; doi:10.3390/ijms241813768)
Supplement: Supplementary file 1 [file ijms-24-13768-s001.zip › Fernandez-Lopez_Supporting Information_Figures S1-S10.pdf]

Supporting Information

Enhancing the Hydrolytic Activity of a Lipase towards Larger Triglycerides through Lid Domain Engineering

Laura Fernandez-Lopez, Sergi Roda, Ana Robles-Martín, Rubén Muñoz-Tafalla, David Almendral, Manuel Ferrer * and Víctor Guallar *

Content

Figure S1……........………........2
Figure S2……...….………........3
Figure S3……...….………...…4
Figure S4……...………......…5
Figure S5……..……...…...…6
Figure S6…….……...…...…7
Figure S7…………...…....…8
Figure S8…….……...…....…9
Figure S9………….......….....10
Figure S10………….....……11

Supplementary Materials

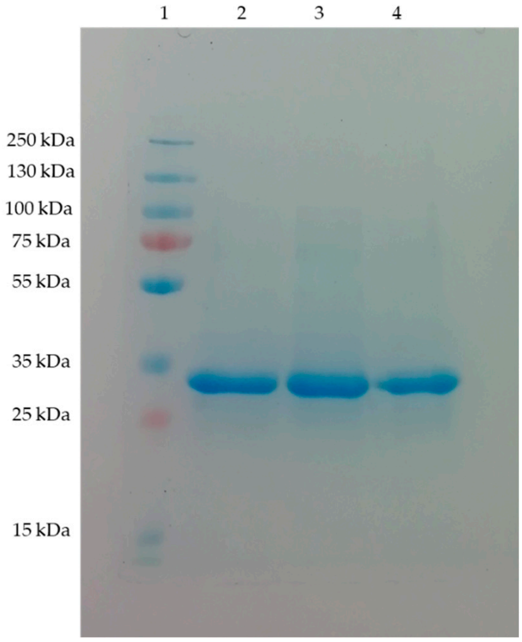

Figure S1. 0.1% SDS–15% PAGE analysis of purified Lip_{MRD}, $\text{Lip}_{\text{MRDlid}}$ and $\text{Lip}_{\text{MRDW89M/L60F}}$. A total of 10 μg protein was used. Lane 1, 10-250 kDa molecular mass marker (Protein marker V (pre-stained), peqGOLD, ref. 27-2210, VWR International bvba, Leuven, Belgium); lane 2, purified Lip_{MRD} ; lane 3, purified $\text{Lip}_{\text{MRDlid}}$; laner 4, purified $\text{Lip}_{\text{MRDW89M/L60F}}$. The image represents an uncropped scans of the gel.

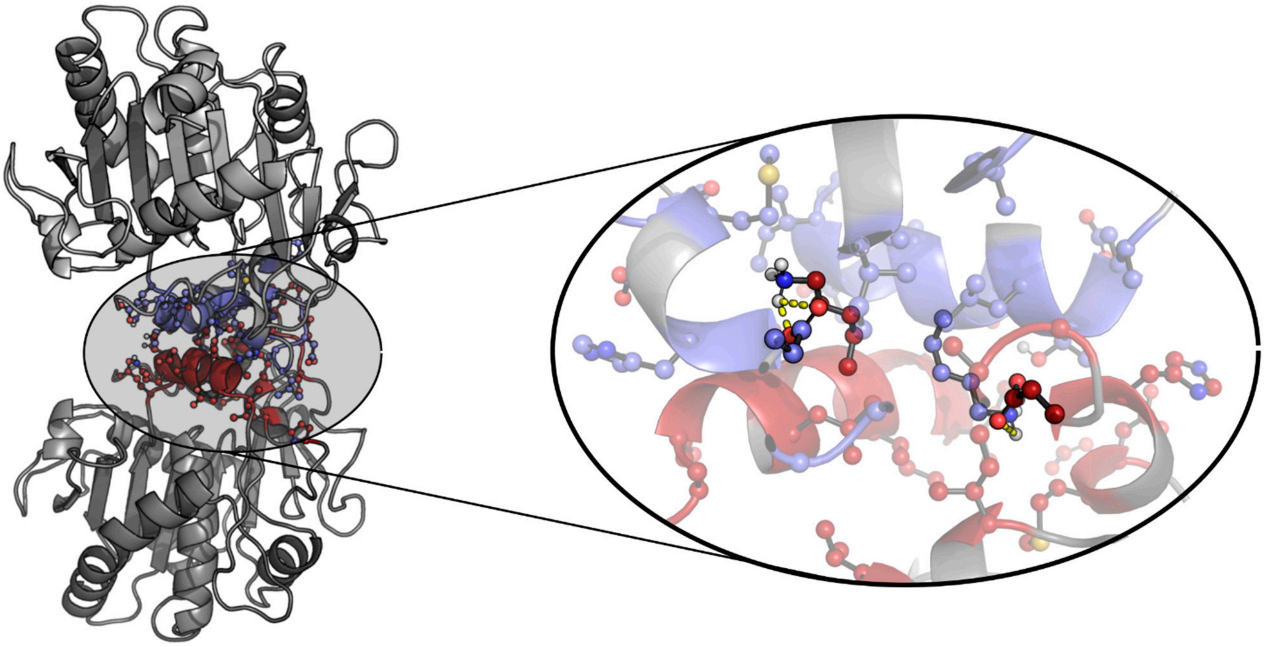

Figure S2. GalaxyHomomer 3D predicted model of a possible dimeric structure of Lip$_{MRD}$. The dimeric interface is highlighted in red and blue for each chain. A zoom to the dimeric interface is displayed to show that the main interaction between monomers would be the salt bridge between K87 and D257.

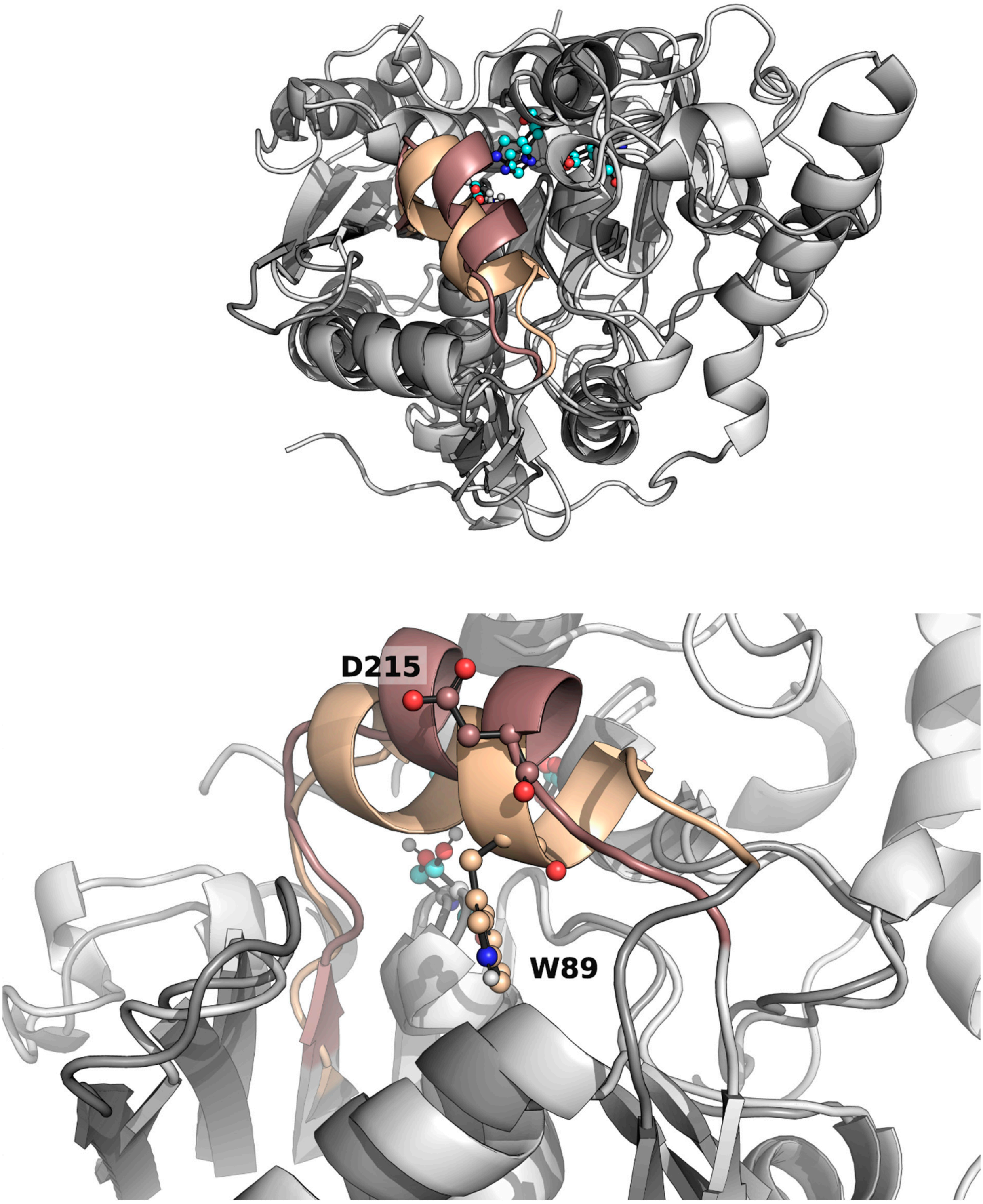

Figure S3. AlphaFold 3D model of *R. delemar* lipase superposed on the LipMRD AlphaFold model highlighting the catalytic triad (with C atoms colored in cyan) and the lid domain (colored in maroon and wheat, respectively) (top). The positions of W89 and D215 in the lid domains of the *R. delemar* and LipMRD lipases are depicted, showing the absence of the bulky W89 residue in the lid domain of the lipase active against long-chain triglycerides (bottom).

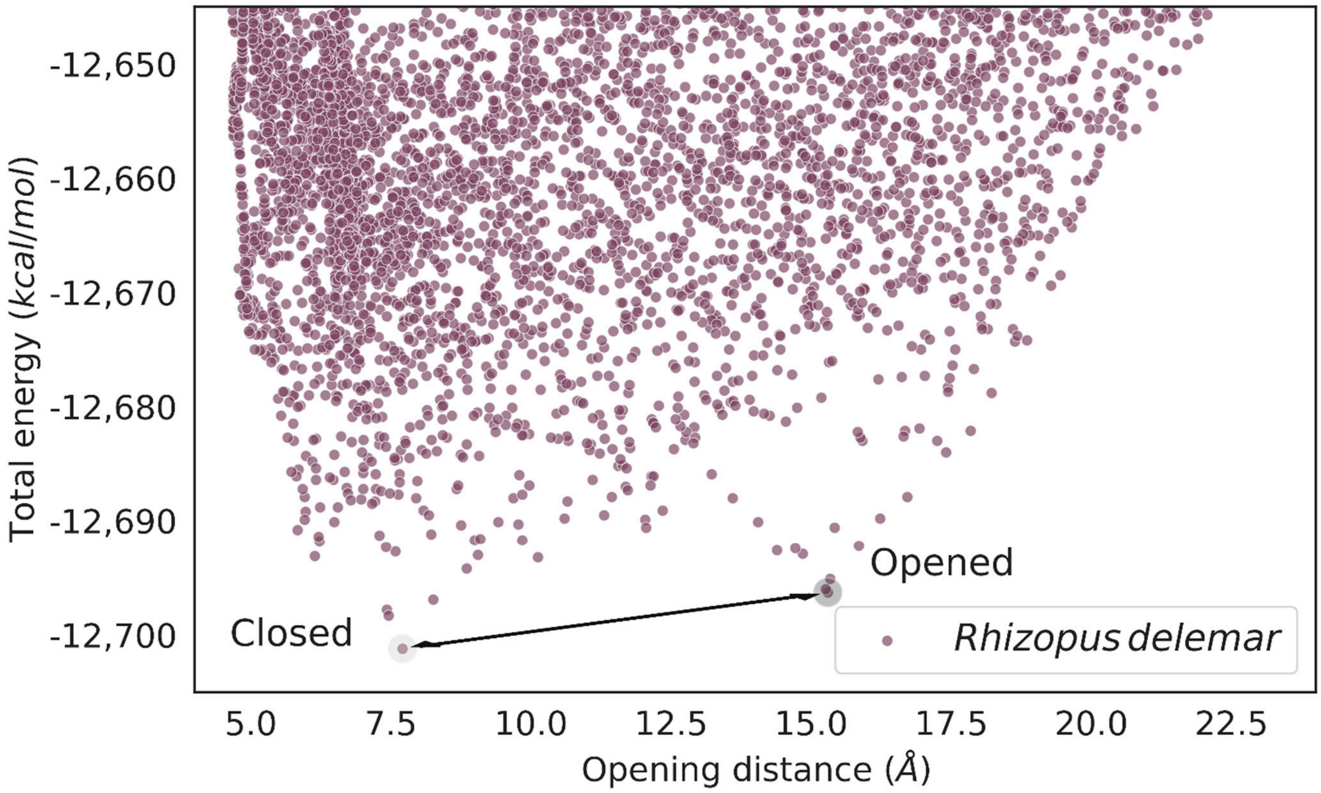

Figure S4. Scatter plots of the opening distance against the total energy of the *R. delemar* lipase. The energy profile was created with the Matplotlib library. The opened and closed metastable states of the *R. delemar* lipase are highlighted.

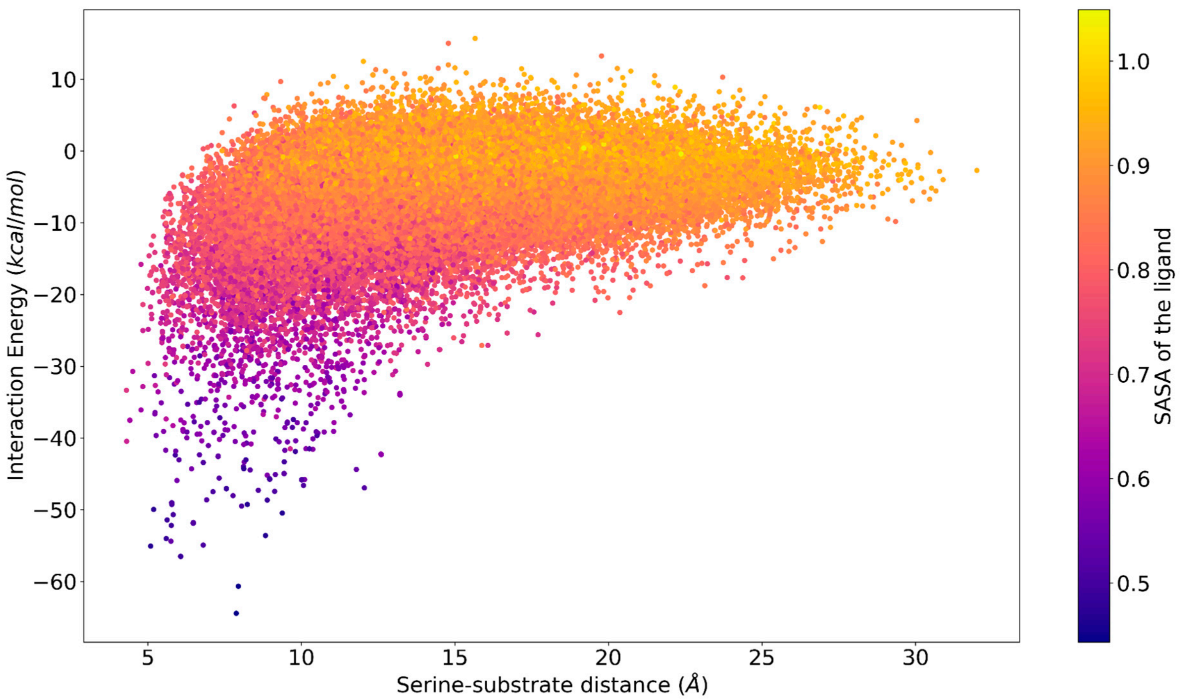

Figure S5. Migration simulation of triolein to LipMRD's active site. The colorbar shows the relative solvent accessible surface area (SASA) of the substrate. The energy profile was created with the Matplotlib library.

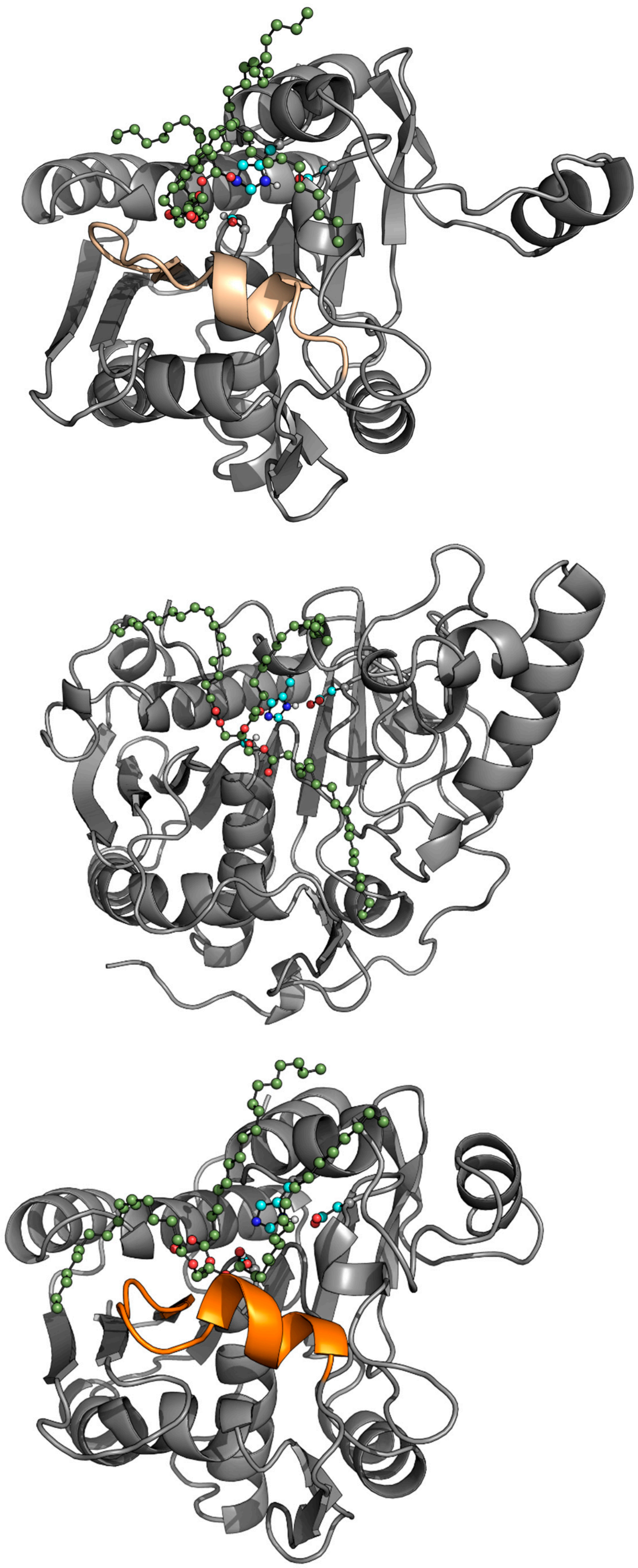

Figure S6. The triolein docking pose used on the open conformation structure (AlphaFold model) of the Lip$_{MRDW89M/L60F}$ double mutant (top), *R. delemar* lipase (middle), and lid-swapped Lip$_{MRDlid}$ mutant (bottom), highlighting the catalytic triad (with C atoms colored in cyan) and the lid domain (colored in wheat for the double mutant, in maroon for the *R. delemar* lipase, and in orange for the lid swapped mutant).

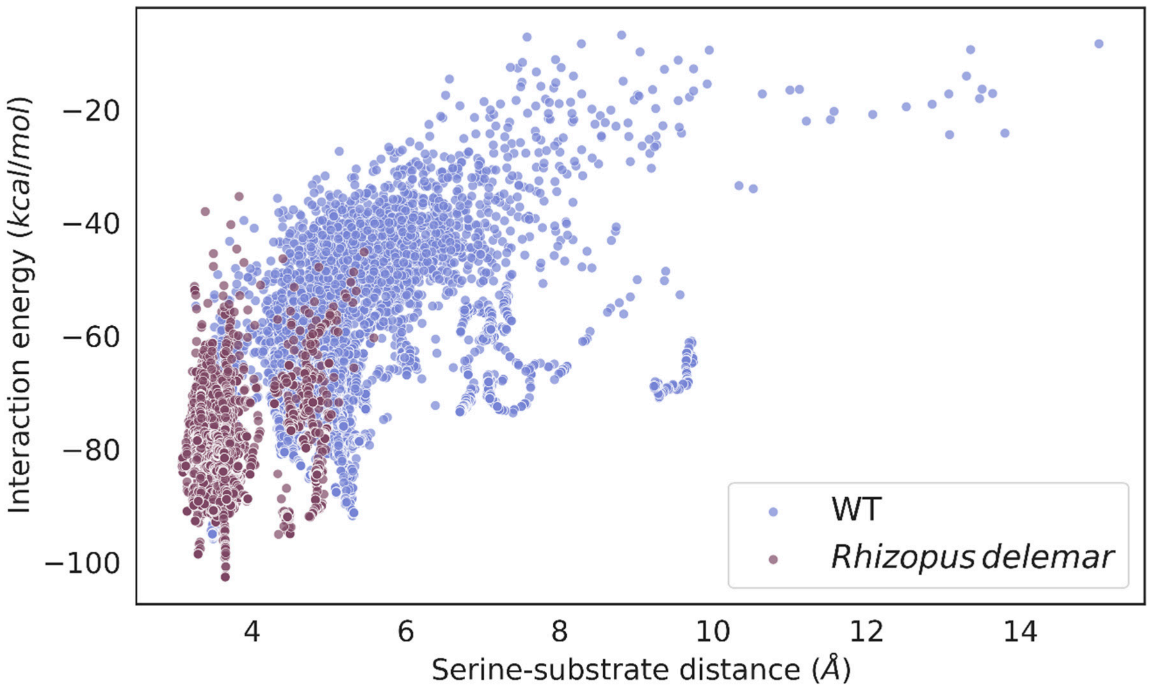

Figure S7. Scatter plots of the serine-substrate (nucleophilic O of the catalytic Ser residue and ester Cs of the substrate) distance against the interaction energy of the wild-type Lip$_{MRD}$ (WT) and *R. delemar* lipases. The energy profiles were created with the Matplotlib library.

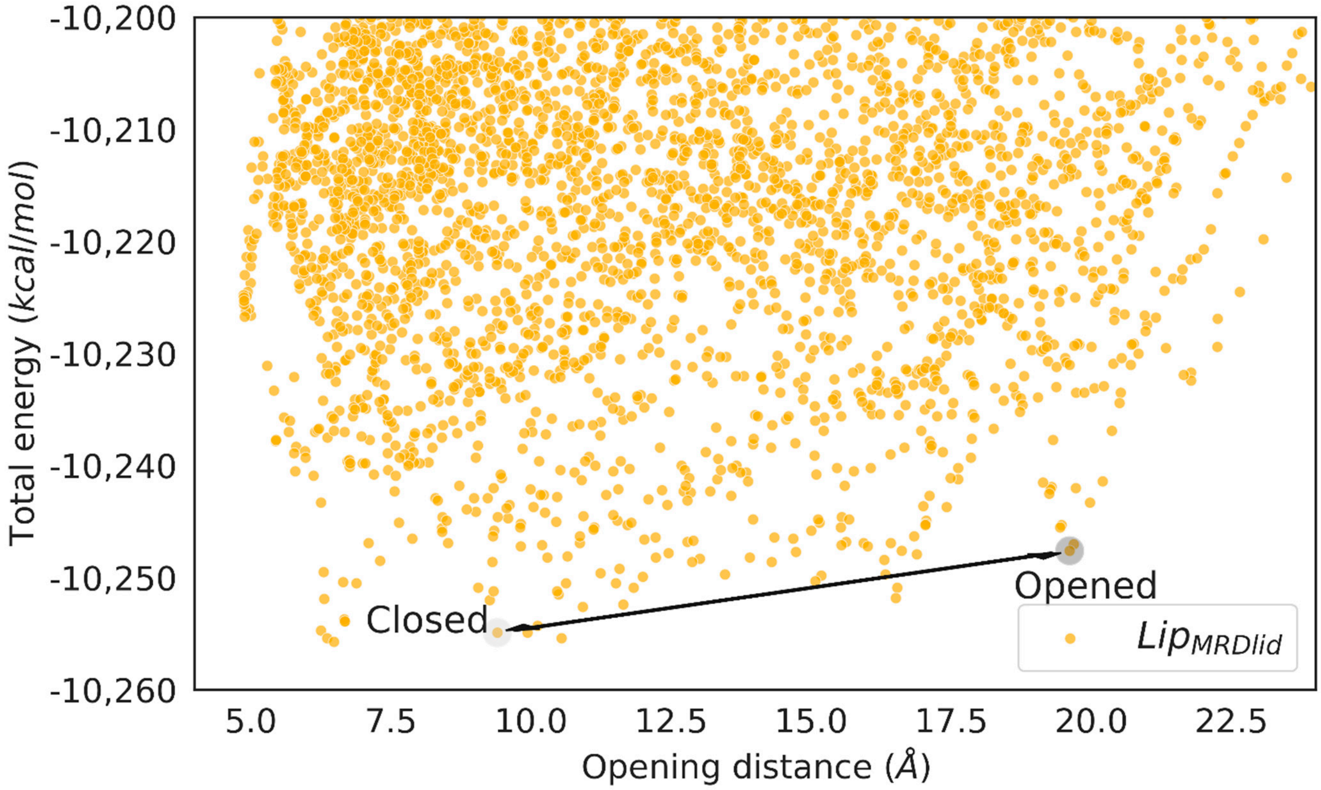

Figure S8. Scatter plots of the opening distance against the total energy of the Lip$_{MRDlid}$ system. The energy profile was created with the Matplotlib library. The opened and closed metastable states of the Lip$_{MRDlid}$ system are highlighted.

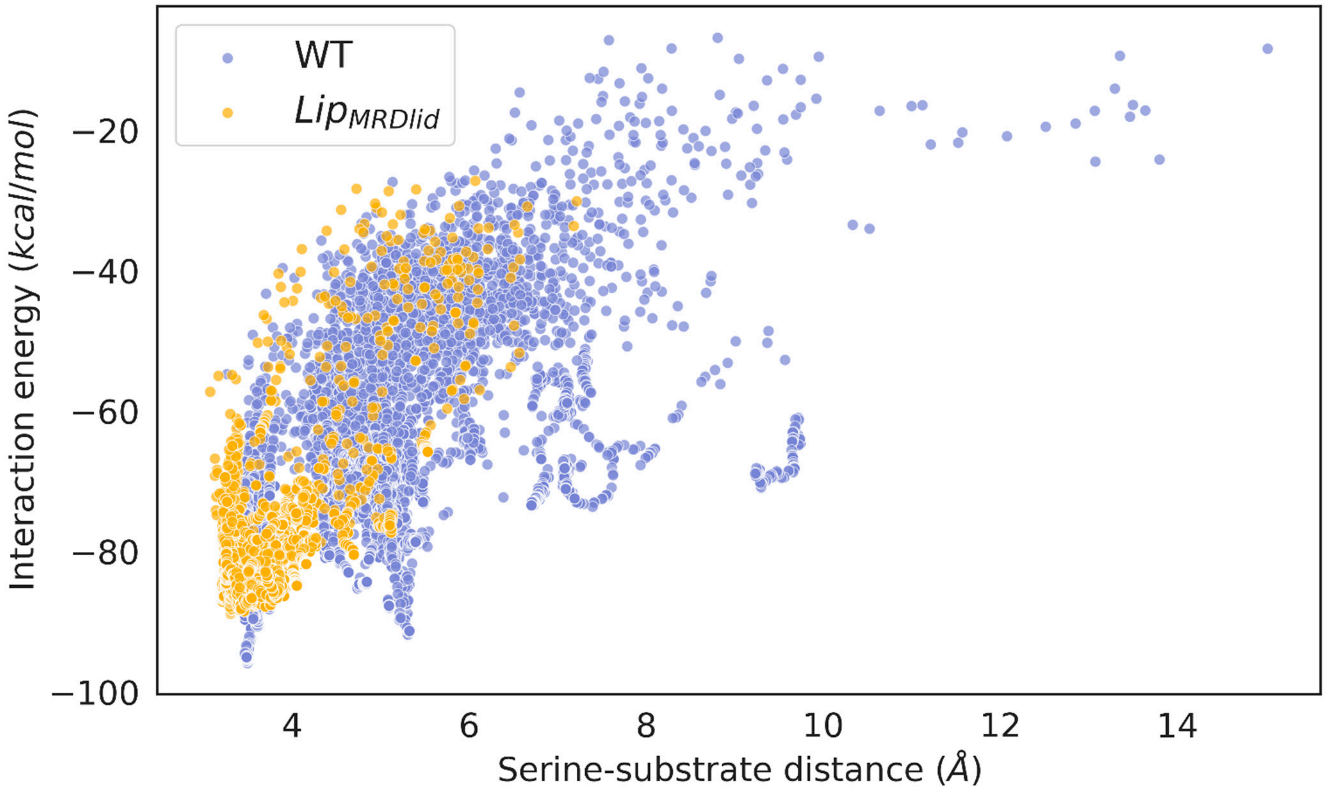

Figure S9. Scatter plots of the serine-substrate (nucleophilic O of the catalytic Ser residue and ester Cs of the substrate) distance against the interaction energy of the wild-type and the lid swapped mutant. The energy profiles were created with the Matplotlib library.

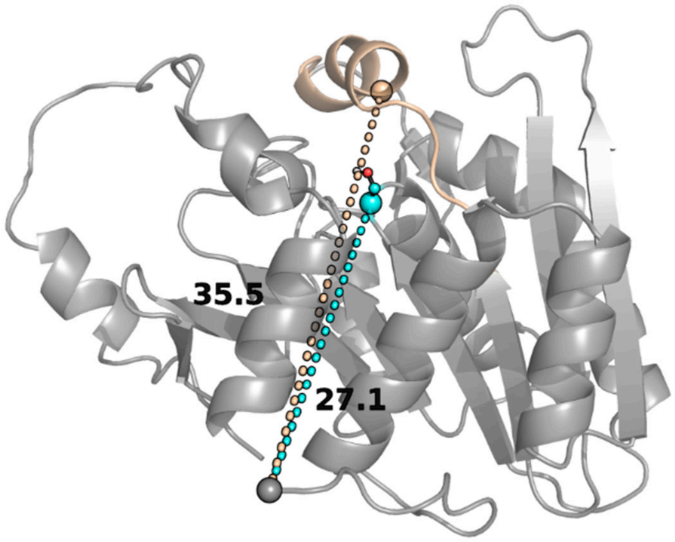

Figure S10. 3D representation of the α carbon distances between the N-terminal residue and the middle residue of the lid domain (colored in wheat), as well as the catalytic serine residue (colored in cyan) in the AlphaFold model.
